# Minimal-invasive Katheterverfahren in der Varizentherapie: Neuer Goldstandard?

**DOI:** 10.1007/s00105-023-05113-w

**Published:** 2023-02-22

**Authors:** Kornelia Böhler

**Affiliations:** Medical University of Vienna, Währinger Gürtel 18–20, 1090 Wien, Österreich

**Keywords:** Varikose, Laserablation, Radiowellenablation, Mechanochemische Ablation, Cyanoacrylatkleber, Varicose vein, Laser ablation, Radiofrequency ablation, Mechanical-chemical ablation, Cyanoacrylate glue

## Abstract

**Hintergrund:**

Katheterverfahren werden in der Therapie der venösen Insuffizienz verbreitet eingesetzt.

**Fragestellung:**

Arten, Funktionsweise und Stellenwert von Katheterverfahren in der Varizentherapie.

**Material und Methode:**

Beschreibung der Anwendung, Wirkungsweise, Risiken und Effizienz von Katheterverfahren anhand von Literaturdaten.

**Ergebnisse:**

Langzeitdaten zur Effizienz bestätigen, dass minimal-invasive Katheterverfahren der offenen Venenchirurgie ebenbürtig sind, bei deutlich geringerer postoperativer Morbidität.

**Schlussfolgerung:**

Minimal-invasive Katheterverfahren sind eine Bereicherung der Therapie venöser Erkrankungen. Die geringe Schmerzbelastung und kurze Ausfallzeiten sind für PatientInnen vorteilhaft.

## Abstract (en)

**Background:**

Endovenous procedures are commonly used for varicose vein treatment.

**Objectives:**

Types, functionality, and significance of endovenous devices.

**Methods:**

To describe the different endovenous devices, their mode of action, inherent risks and efficacy according to the literature.

**Results:**

Long-term data confirm that endovenous procedures are equally effective as open surgery. Postoperative pain is minimal and downtime shorter after catheter interventions.

**Conclusion:**

Catheter-based endovenous procedures increase the diversity of varicose vein treatment options. They are preferred by patients due to less pain and shorter downtime.

Im 20. Jh. galt die Stripping-Operation als Goldstandard in der Therapie der Stammvarikose. Eine zunehmende Hinwendung zu minimal-invasiven Katheterverfahren kann seit der Jahrtausendwende beobachtet werden. Aktuell sind verschiedene Kathetersysteme mit unterschiedlichen Wirkmechanismen auf dem Markt; diese ermöglichen eine individuelle Therapie refluxiver epifaszialer Venen. Die Abkehr von der offenen Chirurgie ist weitgehend vollzogen. Eine profunde Kenntnis der derzeit verfügbaren Katheterverfahren ist elementar, um das passende Verfahren für jeden Patienten auswählen zu können.

## Hintergrund

Bereits zu Beginn des 20. Jh. führte Babcock das intravasale Stripping ein. Wenig später von Navaro um die Krossektomie erweitert, hielt sich die Methode unangefochten als Goldstandard der Varizentherapie, trotz hoher Rezidivraten [17]. Eine neue Ära in der Therapie der Stammvarikose trat mit der Jahrtausendwende ein, als erste Behandlungserfolge mit thermischen Kathetern berichtet wurden [12]. Bemerkenswert ist, dass mit dem Paradigma, die exakte Krossektomie wäre der Schlüssel zu Rezidivfreiheit, gebrochen und das Hauptaugenmerk auf den Verschluss der Stammvene gelegt wurde. Logisch nachvollziehbar wird die therapeutische Konzentration auf den Hauptstamm, wenn man bedenkt, dass die Rezidivgefahr nach Krossektomie ohne Stripping zumindest doppelt so hoch ist wie nach einer Krossektomie mit dem Stripping der Vena saphena magna (VSM; [5]). Voraussetzungen für diesen gewaltigen Entwicklungssprung waren, dass einerseits die Duplexsonographie als basisdiagnostisches Verfahren unter Phlebolog:innen etabliert war, und dass eine allgemeine Tendenz zu minimal-invasiven Verfahren in der Chirurgie bestand. Während die ersten Kathetersysteme durchwegs auf eine thermische Schädigung der Gefäßwand ausgerichtet waren, verfügen wir heute über eine große Auswahl thermischer und nichtthermischer Katheter, die eine individuell angepasste Versorgung von Patienten ermöglichen.

## Allgemeines zur Technik

Alle Katheterverfahren, die zur Therapie der venösen Insuffizienz eingesetzt werden, haben einige Gemeinsamkeiten. Sie werden unter sonographischer Kontrolle peripher über eine Schleuse oder einen Verweilkatheter eingeführt und die Katheterspitze unter Einhaltung eines Sicherheitsabstandes von 1–2 cm unter der Crosse platziert. Alle thermischen Verfahren erfordern eine lokale Anästhesie im gesamten Gefäßverlauf, nichtthermische Verfahren lediglich an der Punktionsstelle. Eine sonographische Kontrolle des postoperativen Verlaufs wird empfohlen. Eine postinterventionelle Kompression wird für thermische und mechanochemische Verfahren empfohlen. Cyanoacrylatkleber erfordern keine Kompression. Ohne adjuvante Therapie der Seitenäste ist eine Kompression für 48 h einer mehrwöchigen Kompression nicht unterlegen [2].

## Thermische Katheter

### Einzelne Verfahren

#### Radiofrequenzablation

Der erste verfügbare Katheter zur Radiofrequenzablation (RFA) war der Closure-Katheter® der Fa. VNUS Medical Technologies, Inc. (Sunnyvale, CA, USA). Sonographisch waren die zentrale kugelförmige Elektrode und der fächerförmige Elektrodenkranz gut zu visualisieren. Um einen direkten Kontakt der Elektroden zur Gefäßwand zu gewährleisten, musste weitgehende Blutleere hergestellt werden, was mithilfe von Lagerungsmanövern, manuellem Druck und perivasaler Tumeszenzlösung gelang. Unter langsamem, kontinuierlichem Rückzug des Katheters wurde die Gefäßwand auf 85 °C erhitzt. Im Rahmen der technischen Weiterentwicklung wurde der Katheter modifiziert und besteht aktuell aus einer ummantelten 7 cm langen Heizspirale (Venefit®/ClosureFast®, Fa. Medtronic, Dublin, Irland). Der Rückzug erfolgt nun segmental mit einer Verweildauer von 20 s/Segment und einer Arbeitstemperatur von 120 °C. In der Crosse-Region und im Bereich dilatierter Segmente wird der Behandlungszyklus 2‑ bis 3‑mal wiederholt. Da die Arbeitstemperatur, Leistung und Einwirkzeit „real time“ am Display des Generators ausgewiesen werden, läuft die Ablation sehr kontrolliert ab. Das Venefit®/ClosureFast®-System ist derzeit das anerkannteste Radiofrequenzverfahren mit dokumentierten 3‑Jahres-Verschlussraten von 92,6 % [18].

Ein bipolares Radiofrequenzverfahren jüngeren Datums ist die radiofrequenzinduzierte Thermotherapie RFiTT® (CelonLab Precision®, Fa. Olympus). Der Katheter trägt an der Spitze 2 durch ein isoliertes Zwischenstück getrennte Elektroden. Der Anordnung der Elektroden zufolge ist die Gefäßwand selbst der Energieleiter und wird direkt auf 60–100 °C aufgeheizt. Nachteilig ist, dass die Rückzugsgeschwindigkeit empirisch über ein akustisches Signal gesteuert wird. Ist eine Elektrode durch einen Thrombus verlegt, wird das System durch die integrierte Autostoppfunktion ausgeschaltet. Die Katheterspitze muss manuell gereinigt und reinseriert werden, um die Ablation fortsetzen zu können.

Die zwei anerkanntesten Radiofrequenzverfahren erzielen 3‑Jahres-Verschlussraten von 92,6 %

Unter den radiowellenbetriebenen Geräten ist auch das EVRF®-Gerät (Fa. F Care Systems, USA) mit dem RF-CR.45i®-Applikator zu erwähnen; dieses wird im Unterschied zu den Vorgenannten mit einer monopolaren Elektrode betrieben. In einem Vergleich der 3 vorgestellten Kathetersysteme lieferten Venefit®, Closure Fast® und RFiTT® nach einem halben Jahr vergleichbare Verschlussraten von 100 % bzw. 98 %, während EVRF mit einer Verschlussrate von 79 % weit zurückfiel [14].

#### Endovenöse Laserablation

Etwa zeitgleich mit dem Closure-Verfahren kamen auch ablative Diodenlaser zur endovenösen Laserablation (EVLA) auf den Markt. Das Energiespektrum lag zu Beginn im niederwelligen Bereich (810 nm, 940 nm, 980 nm), im Bereich des Hämoglobins. Die thermische Schädigung der Gefäßwand erfolgte indirekt über erhitztes Blut. Die aktuelle Lasergeneration arbeitet mit langwelligem Licht (1064 nm, 1320 nm, 1470 nm, 1500 nm). Hauptangriffspunkt ist nicht Hämoglobin, sondern unmittelbar die Gefäßwand. Bei den Laserfasern der ersten Generation erfolgte die Energieabgabe punktuell an der Laserspitze („bare fibre“), weshalb die Einhaltung einer Sicherheitsdistanz von 1–2 cm zur Crosse elementar war, um einer Schädigung der Femoralvene vorzubeugen. Die bare fibre wurde durch Radialsonden (ELVeS® Radial® 2 ring und ELVeS® Radial® 2 ring Pro, ELVeS ®Radial® Slim, ELVeS® Radial® Swift) ersetzt, die die Energie in Form eines Einfach- oder Doppelrings radial abstrahlen. Erhebliche Schwankungen in den angegebenen Behandlungsparametern ergeben sich aufgrund der Vielzahl an unterschiedlichen Lasern mit unterschiedlichen Wellenlängen, aber auch aus individuellen Unterschieden in Bezug auf die eingesetzte Leistung zwischen 10 und 20 W, den Energieabgabemodus, gepulst (1,5 s Pulsdauer/1,5 s Pause) oder kontinuierlich, und die Rückzugsgeschwindigkeit der Laserfaser.

Der Trend bei der endovenösen Laserablation geht in den langwelligen Bereich

Eine nachhaltige thermische Schädigung wird erzielt, wenn Leistung und Rückzugsgeschwindigkeit in Abhängigkeit vom Gefäßdurchmesser so gewählt werden, dass eine querschnittbezogene Energiedichte („endovenous fluence equivalent“, EFE) > 20 J/cm^2^ bzw. eine lineare Energiedichte („linear endovenous energy density“, LEED) von 60–80 J/cm erreicht wird [19]. Unter den langwelligen Lasern hat sich vorerst der 1470 nm Laser durchgesetzt. Der Gefäßverschluss ist zuverlässig, die postoperative Schmerzbelastung dank der Radialfaser deutlich reduziert [15]. Der Trend geht immer weiter in den langwelligen Bereich. So weist ein rezenter Review zur Effizienz von Lasern > 1900 nm 2‑Jahres-Verschlussraten von 96 % aus [24].

#### Heißer Wasserdampf („steam vein sclerosis“)

Die Energiequelle ist heißer Wasserdampf mit Temperaturen bis 120 °C, der pulsatil über einen Katheter appliziert wird. Verdoppelt man die vom Hersteller empfohlene Pulsrate, sind die 2‑Jahres-Verschlussraten der Lasertherapie nicht unterlegen [27]. Im deutschsprachigen Raum hat sich die Methode nicht durchgesetzt.

### Indikationen

Indikationen für thermisch ablative Katheter sind die Stammvarikose der VSM, der Vena saphena parva (VSP), der Vena saphena accessoria anterior (VSAA) und axiale Rezidivgefäße nach Stripping [13]. Die Ablation von Perforansvenen ist technisch herausfordernd, aber möglich. Venöse Ulzera kommen bei frühzeitiger Ablation refluxiver Stammvenen signifikant rascher zur Abheilung [7]. Der Gefäßdurchmesser ist keine limitierende Größe. In einer kleinen, aber sorgfältig dokumentierten Studie konnte gezeigt werden, dass mündungsnahe Aneurysmen > 20 mm bzw. > 15 mm der VSM bzw. VSP mit thermischen Kathetern erfolgreich und nachhaltig verschlossen werden können [9].

### Postoperativer Verlauf

Die postoperative Schmerzbelastung ist deutlich geringer als nach konventioneller Chirurgie und für Radialsonden und segmentale Radiofrequenzkatheter vergleichbar. Eine rasche Rückkehr in den Arbeitsprozess ist die Regel. Bare-fibre-Katheter sind deutlich traumatisierender, mit z. T. der Chirurgie vergleichbaren postoperativen Schmerzen und Hämatomen. An allgemeinen Nebenwirkungen werden Thrombophlebitiden, Hämatome, Hyperpigmentierungen beobachtet. Parästhesien des N. saphenus sind selten und können durch die Wahl des Gefäßzugangs vermieden werden. Etwas gefährdeter ist der N. suralis bei Ablation der VSP. Die perivasale Infiltration von Tumeszenz- oder Kochsalzlösung schützt sowohl vor Nervenläsionen als auch Hautverbrennungen. Wundinfektionen sind kein reales Risiko.

Im zeitlichen Ablauf führt die Anwendung thermischer Katheter unmittelbar zu einer Schrumpfung der Gefäßwand und Thrombosierung des Restlumens. Dieser initiale Schrumpfungseffekt ist nach RFA ausgeprägter als nach EVLA. Closure-Katheter und Radialsonden verursachen homogene und symmetrische, Bare-fiber-Katheter asymmetrische transmurale Schäden und Gefäßperforation. Duplexsonographische Verlaufsbeobachtungen zeigen, dass die VSM ein Jahr postoperativ bei 96 % der Behandelten nicht nachweisbar ist oder sich als hypo- bzw. hyperechogener Strang darstellt [25]. Kommt es zur Rekanalisation, kann langfristig ein Rezidivreflux nicht ausgeschlossen werden.

### Effizienz und Risken

Hinsichtlich ihrer Effektivität sind RFA und EVLA vergleichbar. Die Verschlussraten der VSM betragen nach einem Jahr zwischen 75 % und 94 % [4, 8, 20] sowie nach 5 Jahren zwischen 82 und 97 % [11, 21, 22, 28]. Die klinischen Rezidivraten nach thermischen Verfahren entsprechen jenen der offenen Chirurgie und liegen in der Größenordnung von 18–50 % [6, 11, 29]. Eklatante Unterschiede finden sich in Bezug auf das Crosse-Rezidiv. Während bei offener Chirurgie eine Neovaskularisation der Crosse den Ausschlag gibt, überwiegt bei thermischen Verfahren der Neoreflux in die VSAA [25].

Klinische Rezidivraten nach thermischen Verfahren entsprechen jenen der offenen Chirurgie

In diesem Zusammenhang stellt sich die Frage nach dem Schicksal der in die Crosse einmündenden Seitenäste, die im Rahmen von Katheterverfahren praktisch unbehandelt bleiben. Duplexsonographische Untersuchungen der Crosse-Region 12 Monate nach EVTA der VSM zeigen bei 59 % der Behandelten offene Seitenäste mit regulären Strömungsverhältnissen. Kommt es zu einem Rezidiv, betrifft dies in erster Linie paraaxiale Venen, im Besonderen die VSAA. Dabei kann es sich sowohl um neu aufgetretene Refluxe handeln, wie auch um präoperativ vorhandene und übersehene oder durch ein Steal-Phänomen in die VSM maskierte Refluxe [3]. Aus der bereits erörterten Anwendungstechnik wird klar, dass der bislang empfohlene Sicherheitsabstand zur Crosse von 1–2 cm ausschlaggebend für das paraaxiale Rezidiv sein dürfte. Durch die weite Verbreitung von Radialsonden zeigt sich zunehmend die Tendenz, den Abstand zur Crosse zu reduzieren, um im Mündungsbereich paraaxiale Venen bei der Ablation mitzuerfassen. Kürzlich wurde eine prospektive Untersuchung, in der die Ergebnisse nach Ablation der VSM mit und ohne („flush ablation“) Sicherheitsabstand verglichen wurden, publiziert. Nach Flush ablation war der Reflux im Saphenastumpf signifikant seltener; in der VSAA wurde die Signifikanz knapp verfehlt [23].

### Endovenöse hitzeinduzierte Thrombose

Eine Kernfrage der thermischer Katheterverfahren fokussiert das Risiko der Propagation der hitzeinduzierten Thrombosen ins tiefe Venensystem [10]. Es weist eine Größenordnung von 0–3 % auf, und tritt innerhalb der ersten 72 h, selten bis zu 4 Wochen postoperativ auf. Nach der derzeit geltenden Klassifikation entspricht eine EHIT im unmittelbaren Mündungsbereich dem Grad I, ein < 50 %iger Verschluss des tiefen Gefäßes einem Grad II, ein > 50 %iger Verschluss einem Grad III und ein kompletter Verschluss einem Grad IV [10].

Die hitzeinduzierte tiefe Venenthrombose (TVT) ist sonographisch echodichter als die spontane TVT, ist in Abhängigkeit von der Ausdehnung asymptomatisch und neigt zu einer raschen Regression. Mögliche Risikofaktoren für die Entstehung der EHIT sind fortgeschrittenes Patientenalter, große Gefäßdurchmesser, männliches Geschlecht und TVT im Vorfeld. Die rezent publizierten Leitlinien empfehlen eine „Wait-and-watch“-Strategie bei EHIT-Grad I, eine systemische Antikoagulation in prophylaktischer oder therapeutischer Dosierung bei EHIT-Grad II sowie ab dem Grad III eine therapeutische Dosis [10]. Wöchentliche Duplexsonographiekontrollen sind bis zur Resolution des Thrombus vorgesehen.

Spontane thrombembolische Ereignisse, unabhängig von einer EHIT, sind im Rahmen von Katheterinterventionen insgesamt selten und können durch eine systemische postoperative Thromboembolieprophylaxe weiter gesenkt werden, ohne ein Blutungsrisiko einzugehen. Dennoch ist die Datenlage für eine klare Empfehlung, wer wie lange therapiert werden soll, nicht valide genug [1].

## Nichtthermische Verfahren

### Mechanochemische Ablation

Die mechanochemische Ablation (MOCA) ist ein hybrides Verfahren, bei dem einerseits ein Metalldraht an der Katheterspitze in Rotationen versetzt und zeitgleich flüssiges Verödungsmittel gespritzt wird. Es ist ein „all in all“ batteriebetriebenes Medizinprodukt, das die Effizienz der Verödung steigern soll. Derzeit steht MOCA synonym für das ClariVein®-Kathetersystem. Der Katheter wird in bekannter Weise über eine Verweilkanüle eingeführt, 1 cm unter der Crosse platziert, sodann die Katheterspitze in Rotation versetzt und nach einem weiteren Zentimeter mit dem Einspritzen von Aethoxysklerol begonnen. Während die Katheterspitze rotiert, muss sie kontinuierlich zurückgezogen werden, um einer Verwringung von Katheterspitze und Gefäßwand vorzubeugen.

Thermische Schäden wie Parästhesien sind bei Anwendung der MOCA ausgeschlossen

Entscheidend für den Behandlungserfolg dürfte die Einhaltung der Rückzugsgeschwindigkeit von 1,5 mm/s sein. Vorteil der Methode ist, dass thermische Schäden wie Parästhesien ausgeschlossen sind, ein Trumpf v. a. im Bereich der VSP. Zudem entfällt die Tumeszenzanästhesie. An die Grenzen stößt die Methode bei großen Gefäßdurchmessern, limitierend ist auch die pro Sitzung maximal applizierbare Menge des Aethoxysklerols. So ist beispielsweise bei einem Gefäßdurchmesser von 11 mm nach 25 cm behandelter Gefäßstrecke das zugelassene Gesamtvolumen von 2 % Aethoxysklerol erreicht. Die unmittelbare Gefäßkontraktion fällt deutlich geringer aus als bei den thermischen Verfahren. Die Lernkurve verläuft eher flach, da das Zusammenspiel aus extrem langsamem Rückzug und Applikation von minimalen Mengen des Aethoxysklerols viel Übung erfordert. Valide Langzeitdaten zu MOCA liegen zurzeit noch nicht ausreichend vor. Randomisierte kontrollierte Studien mit einer Laufzeit von 2 und 3 Jahren zeigen, dass die anatomische Verschlussrate nach MOCA geringer ist als nach thermischen Verfahren (77–82 % vs. 91–100 %), dass aber die Verbesserung der klinischen Beschwerden und der Lebensqualität bei beiden Verfahren vergleichbar ist [26].

### Cyanoacrylatkleber

Cyanoacrylatkleber, auch als Superkleber bekannt, wurden erstmals 2012 unter dem Namen VenaSeal® (Fa. Medtronic, CH) für den therapeutischen Einsatz bei peripherer venöser Insuffizienz zugelassen. In der Zwischenzeit sind weitere Systeme auf den Markt gekommen, wie VenaBlock™ oder Veinofff™. Vorteilhaft ist, dass eine postoperative Kompression nicht erforderlich ist. Nachteilig sind protrahierte inflammatorische Reaktionen, die bei 10–20 % der Behandelten beobachtet werden. Im Unterschied zu den thermischen Verfahren findet sich über Jahre hyperechogenes Material im Gefäßlumen. Nur in einzelnen Fällen wurde eine Gefäßresorption berichtet. Die Okklusionsraten betragen nach 36 Monaten 90–95 %. Langzeit-Follow-up-Daten stehen noch aus. In einem Konsensusdokument des Australasian College of Phlebology wird empfohlen, Cyanoacrylatkleber bei Patienten mit chronisch entzündlichen, Autoimmun- und granulomatösen Erkrankungen nicht anzuwenden [16].

## Fazit für die Praxis

- Katheterverfahren haben ihren fixen Platz im therapeutischen Spektrum der Varizenchirurgie. Der Begriff Goldstandard ist zu apodiktisch und in Anbetracht der vergleichbaren Effizienz der chirurgischen und interventionellen Methoden nicht angebracht.
- Aufgrund der Vorteile, die sich aus ambulanter Anwendung, geringer Schmerzbelastung und kurzer Erholungsphase ergeben, werden Patientinnen Katheterverfahren präferieren.
- Bei chirurgisch tätigen Phlebolog:innen ist die Vielfalt der minimal-invasiven Katheter willkommen. Sie ermöglichen eine individuelle Therapie unter Berücksichtigung des spezifischen Krankheitsbildes, der anatomischen Verhältnisse, von Alter und Morbidität sowie der persönlichen Bedürfnisse der Erkrankten.
